# Phenotypic and genomic analysis of bacteria from war wounds in Dnipro, Ukraine

**DOI:** 10.1093/jacamr/dlae090

**Published:** 2024-06-13

**Authors:** Dmytro Stepanskyi, Oksana Ishchenko, Ting Luo, Francois Lebreton, Jason W Bennett, Iryna Kovalenko, Patrick McGann

**Affiliations:** Department of Microbiology, Virology, Immunology, Epidemiology and Biomedical Physics and Informatics, Dnipro State Medical University, Dnipro, Ukraine; Department of Microbiology, Virology, Immunology, Epidemiology and Biomedical Physics and Informatics, Dnipro State Medical University, Dnipro, Ukraine; Multidrug-Resistant Organism Repository and Surveillance Network, Walter Reed Army Institute of Research, Silver Spring, MD, USA; Multidrug-Resistant Organism Repository and Surveillance Network, Walter Reed Army Institute of Research, Silver Spring, MD, USA; Multidrug-Resistant Organism Repository and Surveillance Network, Walter Reed Army Institute of Research, Silver Spring, MD, USA; Department of Microbiology, National Pirogov Memorial Medical University, Vinnytsia, Ukraine; Multidrug-Resistant Organism Repository and Surveillance Network, Walter Reed Army Institute of Research, Silver Spring, MD, USA

## Abstract

**Objectives:**

To better understand the source and potential transmission routes of antibiotic-resistant bacteria infecting injured service members in Ukraine.

**Methods:**

Phenotypic and genomic characterizations were performed on 11 Gram-negative pathogens cultured from war wounds at an intermediate evacuation hospital in Dnipro.

**Results:**

The analysis revealed both susceptible and extensively drug-resistant strains present in cultures, including high-risk global clones carrying carbapenemases.

**Conclusions:**

Globally distributed carbapenemase-producing lineages are being acquired early in the medical evacuation process.

## Introduction

Before the full-scale invasion by the Russian Federation in February 2022, the prevalence of MDR organisms (MDROs) in Ukraine was estimated at 19.5%.^1^ However, this rate varied significantly among species, with a 2022 WHO/ECDC study of Ukraine indicating that carbapenem resistance varied from 4% in *Escherichia coli* to 53.3% in *Klebsiella pneumoniae* in 2020 (https://www.ecdc.europa.eu/sites/default/files/documents/Joint-WHO-ECDC-AMR-report-2022.pdf) Recently, a Ukrainian-led international collaboration identified multiple challenges in controlling MDROs and healthcare-associated infections (HAIs), including inadequate surveillance and infection prevention,^2^ but the onset of the invasion has greatly exacerbated the problem.^3^ Notably, these observations reflect previous trends from armed conflicts in Afghanistan, Syria^4^ and Iraq,^5^ where MDRO infections increased significantly in military and civilian populations.

Several studies have provided insights into the species and antibiotic susceptibilities of bacteria causing war wound infections in Ukraine.^3,6–9^ These have revealed multiple species, especially *Acinetobacter baumannii*, *K. pneumoniae* and *Pseudomonas aeruginosa* carrying an array of antimicrobial resistance (AMR) genes. Critically, these bacteria were obtained from patients near the end of the evacuation process, obscuring their origins and potential transmission routes. In this study, we provide further insights into the epidemiology of these bacteria by performing phenotypic and genomic characterizations of 11 Gram-negative bacteria causing infectious complications of combat-related injuries in Dnipro, an intermediate evacuation hub for war-wounded.

## Materials and methods

Three *E. coli*, three *K. pneumoniae* and five *P. aeruginosa* were cultured from 11 injured Ukrainian service members between December 2022 and March 2023 at a hospital in Dnipro (Table 1). Patients were selected based on the severity of trauma and infectious complications from combat injuries with chronic non-healing wounds. Antibiotic susceptibility testing (AST) was performed on site by disc diffusion using EUCAST guidelines (V14.0, 2024; www.eucast.org). In addition, all isolates were sent to the Multidrug Resistant Organism Repository and Surveillance Network (MRSN) in the USA, where AST was repeated on a VITEK 2 system, and additional testing performed using broth microdilution or a customized Sensititre plate, as applicable. For cefiderocol testing, broth microdilution was performed in iron-depleted cation-adjusted Mueller–Hinton broth as recommended by the CLSI. All results were interpreted using CLSI guidelines (Table S1, available as Supplementary data at *JAC-AMR* Online). Finally, WGS was performed on a MiSeq benchtop sequencer, followed by high-resolution genomic analysis, as previously described.^8^

**Table 1.** Characteristics of the 11 war wound isolates in this study

MRSN ID	Species	ST^a^	Date^b^	Antimicrobial resistance genes^c^
122283	*E. coli*	131	12/2022	*aac(3)-IId*, ***bla*_CTX-M-15_**, *bla*_TEM-1_
122311	*E. coli*	46	3/2023	*aadA2*, ***bla*_CTX-M-15_**, ***bla*_NDM-5_**, *dfrA12*, *erm(B)*, *mph(A)*, *sul1*
122312	*E. coli*	8184	3/2023	ND
122259	*K. pneumoniae*	395	5/2023	*aac(3)-IIe*, ***aac(6′)-Ib-cr5***, *aph(3′)-VI*, ***armA***, ***bla*_CTX-M-15_**, ***bla*_NDM-1_**, *bla*_OXA-1_, *bla*_SHV-1_, *bla*_TEM-1_, *catA1*, *catB3*, *dfrA5*, *fosA*, *mph(A)*, *msr(E)*, *qnrS1*, *sul1*, *sul2*
122289	*K. pneumoniae*	392	1/2023	*aph(3′′)-Ib*, *aph(6)-Id*, ***bla*****_CTX-M-15_**, *bla*_SHV-11_, *bla*_TEM-1_, *dfrA14*, *fosA*, *qnrB1*, *sul2*
122313	*K. pneumoniae*	23	3/2023	*bla*_SHV-11_, *fosA*
122260	*P. aeruginosa*	1047	1/2023	*aac(6′)-Ib3*, *ant(2′′)-Ia*, *aph(3′′)-Ib*, *aph(3′)-IIb*, *aph(6)-Id*, ***bla*****_IMP-1_**, *bla*_OXA-10_, *bla*_OXA-488_, *bla*_PDC-12_, *catB7*, *dfrA1*, *fosA*, *qnrVC1*, *sul1*
122285	*P. aeruginosa*	773	12/2022	*aadA11*, *aph(3′)-IIb*, ***bla*****_NDM-1_**, *bla*_OXA-395_, *bla*_PDC-16_, *catB7*, *fosA*, *qnrVC1*, ***rmtB4***, *sul1*, *tet(G)*
122298	*P. aeruginosa*	773	2/2023	*aadA11*, *aph(3′)-IIb*, ***bla*****_NDM-1_**, *bla*_OXA-395_, *bla*_PDC-16_, *catB7*, *fosA*, *qnrVC1*, ***rmtB4***, *sul1*, *tet(G)*
122309	*P. aeruginosa*	428	3/2023	*aph(3′)-IIb*, *bla*_OXA-494_, *bla*_PDC-8_, *catB7*, *fosA*
122310	*P. aeruginosa*	428	3/2023	*aph(3′)-IIb*, *bla*_OXA-494_, *bla*_PDC-8_, *catB7*, *fosA*

ND, none detected.

^a^ *In silico*-derived multi-locus sequence type.

^b^Month and year isolate was cultured.

^c^ *In silico*-derived antimicrobial resistance gene content generated using MIGHT, a customized script wrapping ARIBA (https://github.com/sanger-pathogens/ariba) and AMRfinder (https://github.com/ncbi/amr). Genes in bold are high impact.

## Results

**Figure 1.**
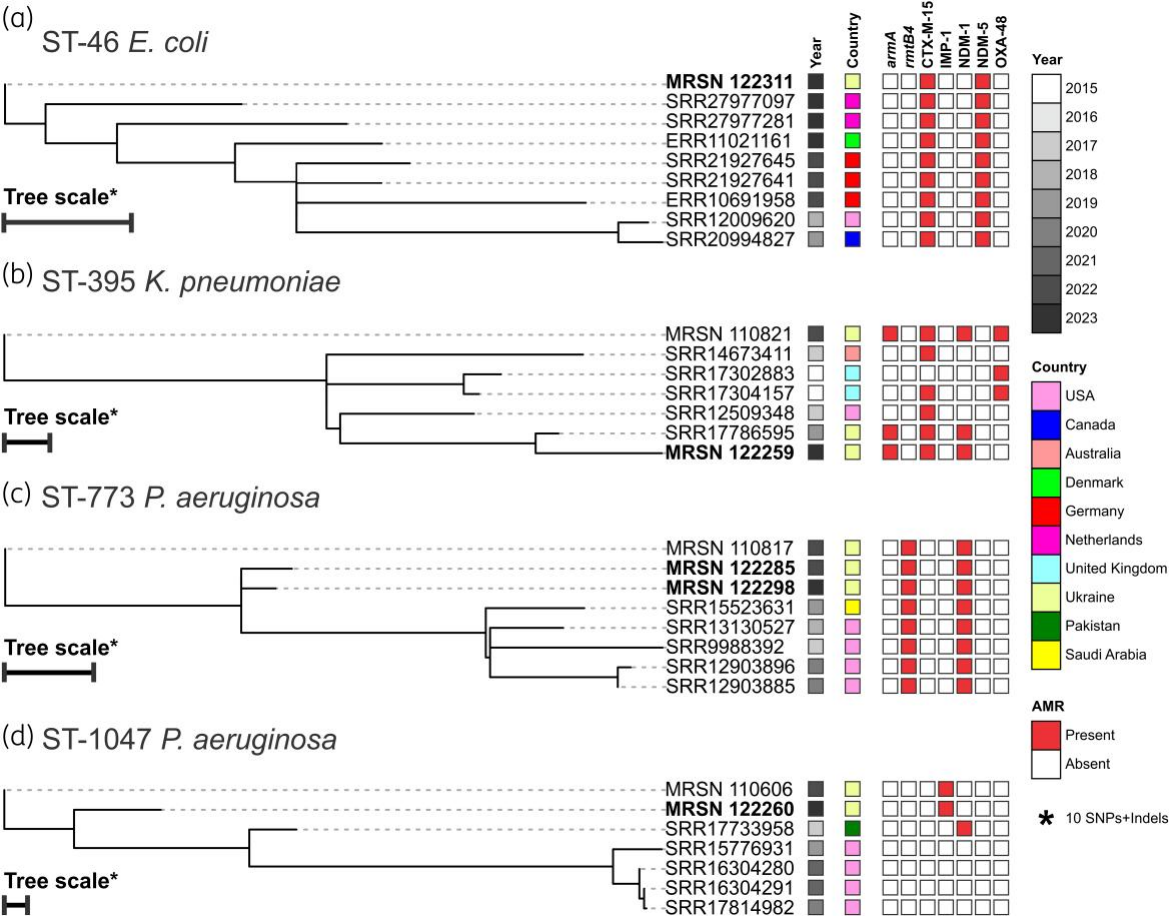
Core genome, SNP-based phylogenetic tree for (a) ST46 *E. coli*, (b) ST395 *K. pneumoniae*, (c) ST773 *P. aeruginosa* and (d) ST1047 *P. aeruginosa*. In addition to the isolates in this study (highlighted in bold), the dataset included the five closest genetic neighbours from the Pathogenwatch repository (https://pathogen.watch/) and the genomes from McGann *et al*.^8^ Year of collection, country of origin, and presence (closed square) or absence (open square) of selected AMR genes are indicated.

The three *E. coli* were assigned to ST46, ST131 and ST8184 and displayed different susceptibility profiles (Table 1, Table S1). Notably, *E. coli* MRSN 122312 (ST8184) was susceptible to all antibiotics tested and carried no AMR genes. *E. coli* MRSN 122283 belonged to Clade A of the globally distributed ST131^10^ and was susceptible to 19 of the 29 antibiotics tested. The phenotype correlated with the presence of three AMR genes, including the ESBL *bla*_CTX-M-15_, and point mutations in *gyrA* (D87N and S83L) and *parC* (E84V and S80I) that confer fluoroquinolone resistance. *E. coli* MRSN 122311 was the most concerning and exhibited susceptibility to just 12 of the 29 antibiotics. Notably, the isolate was resistant to both imipenem and meropenem and to cefiderocol (Table S1). Resistance to the carbapenems could be traced to the presence of *bla*_NDM-5_. Similarly, cefiderocol resistance was likely due to the presence of *bla*_NDM-5_ and mutations resulting in a non-functional OmpC and a four amino acid substitution (YRIN) at position 333 of penicillin-binding protein 3 (PBP3) in these isolates, as previously described.^11,12^ The isolate belonged to ST46, a lineage recently implicated in the expansion of *E. coli* carrying the *bla*_NDM-5_ carbapenemase across Europe.^13^ Similar to those isolates, MRSN 122311 carried *bla*_CTX-M-15_, and a comparison with ST46 genomes available on EnteroBase (enterobase.warwick.ac.uk) revealed a high genetic relatedness (5–51 SNPs) to isolates cultured from patients in Denmark, Germany and the Netherlands (Figure 1a). Limited data are available at the NCBI on these isolates, but the three German isolates and the one Danish isolate were cultured from Ukrainian refugees receiving care in those countries.

The three *K. pneumoniae* belonged to three different STs, ST23, ST392 and ST395 (Table 1) with distinct antibiotic susceptibility profiles (Table S1). MRSN 122313 was susceptible to 27 of the 28 antibiotics but belonged to the well-known hypervirulence lineage ST23 (KL1), had a Kleborate score^14^ of 5, and carried all five virulence markers that best predict a hypervirulent phenotype (*iucA*, *iroB*, *peg-344*, *rmpA* and *rmpA2*).^15^ In contrast, the ST392 (KL27) MRSN 122289 was an ESBL-producer (*bla*_CTX-M-15_), was susceptible to 19 of the 28 antibiotics tested, had a Kleborate score of 1, and only carried the *ybt16* gene (encoding a yersiniabactin siderophore) on a chromosomal ICE*Kp12*. This lineage is globally distributed and has been linked to infections in humans and companion animals.^16^ Finally, MRSN 122259 was susceptible to just 7 of the 28 antibiotics tested and, similar to *E. coli* MRSN 122311, was also resistant to cefiderocol and the carbapenems. Carbapenem resistance was conferred by *bla*_NDM-5_, which also contributed to the cefiderocol resistance, and the presence of a mutation leading to a truncated OmpK35, as previously described.^11,17^ The isolate belonged to the globally emerging carbapenemase-producing lineage ST395 (KL2KL30).^18^ Similar to other ST395 isolates, it carried the 16S methyltransferase (RMTase) *armA*, *bla*_NDM-5_ and *bla*_CTX-M-15_ (Table 1). Notably, the isolate had a Kleborate^14^ virulence score of 4 and carried four of the five hypervirulence markers (missing *iroB*). When compared with other ST395 isolates, it clustered with recent Ukrainian isolates (27–104 SNPs) and historical strains from the USA, the UK and Australia (Figure 1b).

The five *P. aeruginosa* were assigned to three separate STs: ST428 (two isolates), ST773 (two isolates) and ST1047 (one isolate) (Table 1). All carried the *P. aeruginosa* intrinsic AMR genes—*aph(3′)-IIb*, *bla*_OXA-10-like_, *bla*_PDC_, *catB* and *fosA*—but antibiotic susceptibility and the presence of acquired AMR genes differed significantly. Both ST428 isolates were susceptible to 17 of the 19 antibiotics tested and carried no additional acquired AMR genes. In contrast, both ST773 isolates were susceptible to just two antibiotics, cefiderocol and colistin (Table S1), and carried the same six acquired AMR genes, including the *bla*_NDM-1_ carbapenemase and the *rmtB4* 16S RMTase (Figure 1c). The isolates were most related to one cultured from an injured Ukrainian soldier in 2023 (15–16 SNPs),^8^ and more distantly (28–40 SNPs) to strains from the USA and Saudi Arabia obtained between 2017 and 2020 (Figure 1c). Isolate MRSN 122260 (ST1047) shared a similar AST profile with the ST773 isolates, except for a significantly elevated MIC of aztreonam/avibactam (32 mg/L versus 4 mg/L; Table S1). The isolate carried nine acquired AMR genes, including the *bla*_IMP-1_ carbapenemase. The isolate was most related (45 SNPs) to an IMP-1-producing strain cultured from the blood of a Ukrainian soldier in 2023 (Figure 1d),^8^ and to an NDM-1-producing strain cultured from a respiratory specimen at the Pakistan Institute of Medical Sciences in 2017 (58 SNPs). Four other ST1047 strains collected in the USA between 2019 and 2021 were more distantly related (45 to 110 SNPs) but lacked a carbapenemase.

## Discussion

Though only a limited number of strains were included in this study, it provides the first snapshot of bacteria being cultured from war wounds soon after injury. In most evacuation cases, the Dnipro region is an intermediate hub on the way to definitive medical care and rehabilitation further west.^19^ However, due to the ongoing war, delayed transfers, crowded hospitals and limited infection control resources the risk of infectious complications and HAIs is increased. As demonstrated in this report, both susceptible and MDR strains are being cultured in Dnipro, indicating that MDR strains are already infecting wounds at this point in the evacuation chain.

## Supplementary Material

dlae090_Supplementary_Data

## Data Availability

Genomes described herein have been deposited at Genbank under Bioproject number PRJNA1090910.

## Supplementary data

Table S1 is available as Supplementary data at *JAC-AMR* Online.
