# Supplementary material for: Phenotypic and genomic analysis of bacteria from war wounds in Dnipro, Ukraine
Source: JAC Antimicrob Resist. 2024 Jun 13;6(3):dlae090. doi: 10.1093/jacamr/dlae090 (PMC11170486; doi:10.1093/jacamr/dlae090)
Supplement: dlae090_Supplementary_Data [file dlae090_supplementary_data.zip › Supplemental_Table_1_-_Minimum_Inhibitory_Concentrations_CLEAN.docx]

**Supplemental Table S1. Minimum Inhibitory Concentrations** **for 11 isolates from Dnipro, Ukraine.**

|  | ***Escherichia coli*** | | | ***Klebsiella pneumoniae*** | | | ***Pseudomonas aeruginosa*** | | | | |
| --- | --- | --- | --- | --- | --- | --- | --- | --- | --- | --- | --- |
| **Sequence Type**^1^ | **131** | **46** | **8184** | **395** | **392** | **23** | **1047** | **773** | **773** | **428** | **428** |
| **Strain ID** | **122283** | **122311** | **122312** | **122259** | **122289** | **122313** | **122260** | **122285** | **122298** | **122309** | **122310** |
|  | **Minimum Inhibitory concentration (mg/L)^2^** | | | | | | | | | | |
| Amikacin (V2) | <=2 | <=2 | <=2 | >=64 | <=2 | <=2 | >=64 | >=64 | >=64 | <=2 | <=2 |
| Ampicillin/sulbactam (V2) | >=32 | >=32 | <=2 | >=32 | >=32 | 4 |  |  |  |  |  |
| Ampicillin (V2) | >=32 | >=32 | 8 |  |  |  |  |  |  |  |  |
| Aztreonam (V2) | 16 | >=64 | <=1 | >=64 | >=64 | <=1 |  |  |  |  |  |
| Aztreonam/Avibactam (BMD) | < 0.0625 | 2 | < 0.0625 | 0.125 | 0.25 | < 0.0625 | 32 | 4 | 4 | 8 | 8 |
| Cefepime (V2) | >=32 | >=32 | <=0.12 | >=32 | >=32 | <=0.12 | >=32 | >=32 | >=32 | 2 | 2 |
| Cefiderocol (BMD) | 2 | 32 | < 0.25 | 16 | 1 | < 0.25 | 4 | 2 | 1 | < 0.25 | < 0.25 |
| Ceftazidime (V2) | 16 | >=64 | <=1 | >=64 | >=64 | <=1 | >=64 | >=64 | >=64 | 4 | 4 |
| Ceftazidime/Avibactam (V2) | <=0.12 | >=16 | <=0.12 | >=16 | 0.25 | <=0.12 | >=16 | >=16 | >=16 | 2 | 2 |
| Ceftolozane/Tazobactam (V2) | <=0.25 | >=32 | <=0.25 | >=32 | 1 | <=0.25 | >=32 | >=32 | >=32 | 1 | 1 |
| Ceftriaxone (V2) | >=64 | >=64 | <=0.25 | >=64 | >=64 | <=0.25 |  |  |  |  |  |
| Ciprofloxacin (V2) | >=4 | >=4 | <=0.25 | >=4 | >=4 | <=0.25 | >=4 | >=4 | >=4 | <=0.25 | <=0.25 |
| Colistin (S) | <= 0.25 | <= 0.25 | <= 0.25 | <= 0.25 | <= 0.25 | <= 0.25 | 2 | 1 | 1 | 0.5 | 2 |
| Eravacycline (S) | 0.25 | 0.25 | 0.12 | 0.25 | 0.5 | 0.5 |  |  |  |  |  |
| Ertapenem (V2) | <=0.12 | >=8 | <=0.12 | >=8 | <=0.12 | <=0.12 |  |  |  |  |  |
| Gentamicin (V2) | >=16 | <=1 | <=1 | >=16 | <=1 | <=1 | >=16 | >=16 | >=16 | <=1 | <=1 |
| Imipenem (V2) | <=0.25 | >=16 | <=0.25 | >=16 | <=0.25 | <=0.25 | >=16 | >=16 | >=16 | 1 | 1 |
| Imipenem/Relebactam (S) | 0.12 | 16 | 0.25 | 16 | 0.25 | 0.12 | 8 | > 16 | > 16 | 0.25 | 0.25 |
| Levofloxacin (V2) | >=8 | >=8 | <=0.12 | >=8 | >=8 | <=0.12 | >=8 | >=8 | >=8 | 0.25 | 1 |
| Meropenem (V2) | <=0.25 | 8 | <=0.25 | 8 | <=0.25 | <=0.25 | >=16 | >=16 | >=16 | 0.5 | 0.5 |
| Meropenem/Vaborbactam (S) | 0.015 | > 16 | 0.015 | > 16 | 0.015 | 0.03 | > 16 | > 16 | > 16 | 0.25 | 0.25 |
| Nitrofurantoin (V2) | <=16 | <=16 | <=16 | >=512 | 64 | 64 |  |  |  |  |  |
| Omadacycline (S) | 1 | 2 | 1 | 2 | 2 | 2 |  |  |  |  |  |
| Piperacillin/Tazobactam (V2) | <=4 | >=128 | <=4 | >=128 | 8 | <=4 | >=128 | >=128 | >=128 | 8 | 8 |
| Plazomicin (S) | 1 | 1 | 1 | > 4 | 0.25 | 0.25 | > 4 | > 4 | > 4 | 4 | 4 |
| Tetracycline (V2) | <=1 | 4 | <=1 | 2 | 2 | <=1 |  |  |  |  |  |
| Ticarcillin/clavulanic acid (V2) |  |  |  |  |  |  | >=128 | >=128 | >=128 | 32 | 64 |
| Tigecycline (V2) | <=0.5 | <=0.5 | <=0.5 | <=0.5 | <=0.5 | <=0.5 |  |  |  |  |  |
| Tobramycin (V2) | 8 | <=1 | <=1 | >=16 | <=1 | <=1 | >=16 | >=16 | >=16 | <=1 | <=1 |
| Trimethoprim/sulfamethoxazole (V2) | <=20 | >=320 | <=20 | >=320 | >=320 | <=20 |  |  |  |  |  |

^1^ *In silico* derived multi-locus sequence type.

^2^ Minimum Inhibitory Concentration (MIC) expressed as µg/ml. MICs were performed using either the Biomerieux Vitek2 (V2) with cards AST-95 and XN-09, a Thermo Scientific Sensititre (S) customized panel, or broth microdilution (BMD) using CLSI Guidelines (REF). Green shading, Susceptible; Orange shading, Non-susceptible (Resistant or Intermediate); Blue shading, No CLSI Interpretations currently available.
