# Supplementary figures and images for: Phenotypic and genomic analysis of bacteria from war wounds in Dnipro, Ukraine
Source: JAC Antimicrob Resist. 2024 Jun 13;6(3):dlae090. doi: 10.1093/jacamr/dlae090 (PMC11170486; doi:10.1093/jacamr/dlae090)

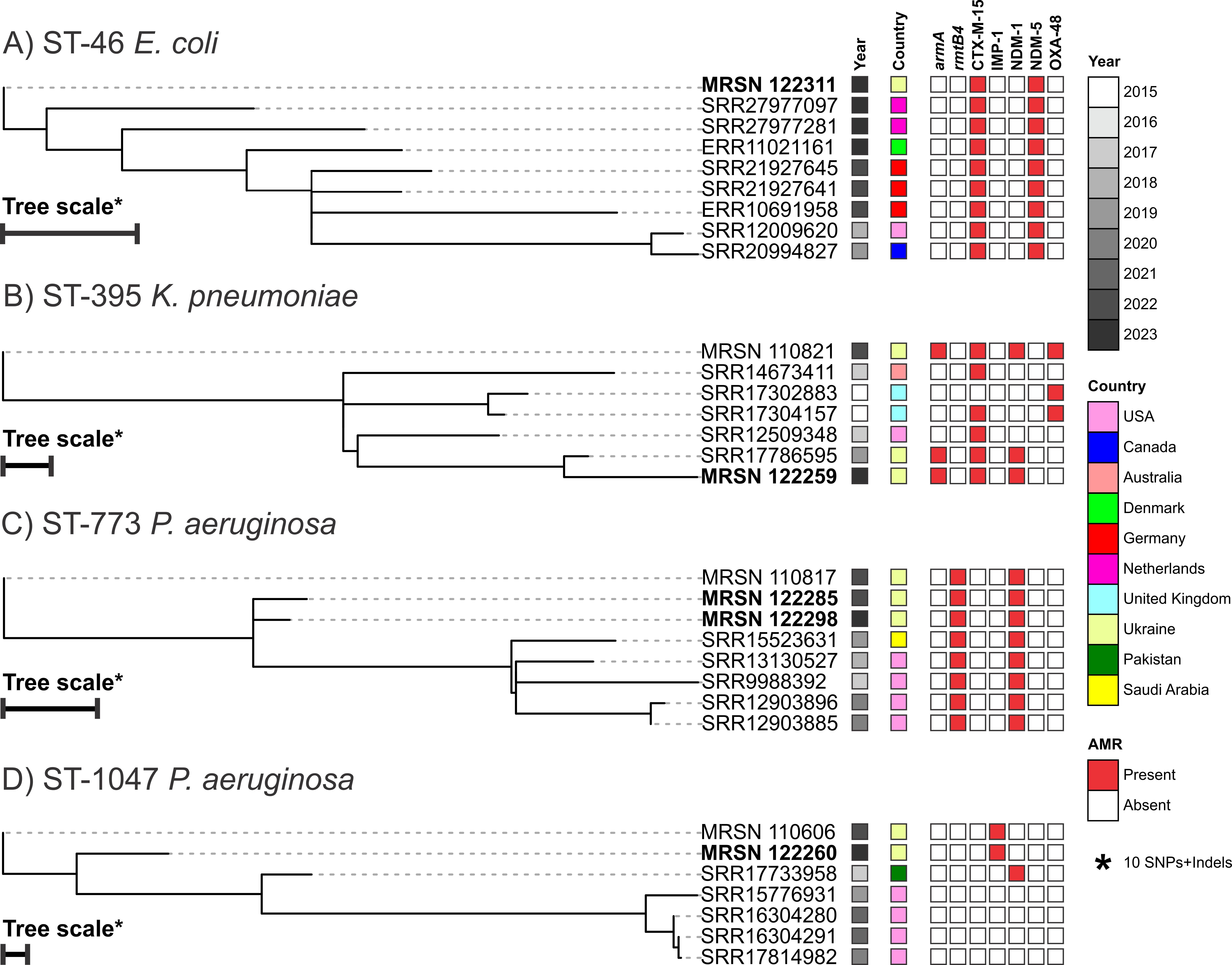

Supplement: dlae090_Supplementary_Data [file dlae090_supplementary_data.zip › Figure 1 - Core genome, SNP-based phylogenetic tree.png]
